# Data on the functional consequences of the mutations identified in 21-Hydroxylase deficient CAH patients

**DOI:** 10.1016/j.dib.2018.05.043

**Published:** 2018-05-19

**Authors:** Ragini Khajuria, Rama Walia, Anil Bhansali, Rajendra Prasad

**Affiliations:** a Department of Biochemistry, Postgraduate Institute of Medical Education and Research, Chandigarh, India; b Department of Endocrinology, Postgraduate Institute of Medical Education and Research, Chandigarh, India

## Abstract

This article presents the data set regarding the functional characterization of mutations in CYP21A2 gene in CAH patients as described in “Functional characterization and molecular modeling of the mutations in *CYP21A2* gene from patients with Congenital Adrenal Hyperplasia (Khajuria et al., 2018) [1]. This data set features about the identification of mutations and their functional characterization by bioinformatic tools (mutation severity prediction softwares). Molecular modeling enabled us to locate the site of the amino-acid residues in 3-Dimensional model of 21-Hydroxylase protein which were mutated in patients.

**Specifications Table**

**Table:** 

Subject area	Biology
More specific subject area	Endocrinology, Bioinformatics
Type of data	Tables, Figures
How data was acquired	DNA sequencing, Swiss-PDB viewer (3D structure), mutation severity prediction software’s
Data format	Analyzed
Experimental factors	DNA from blood of CAH patients
Experimental features	Bioinformatic tools
Data source location	Chandigarh, India
Data accessibility	NA

**Value of the data**

• The 3-Dimensional structure of the 21-Hydroxylase protein illustrates the site of the amino acid residues found to be mutated in cohort under study.
• The data indicated the sequence of the primers constructed for the functional characterization of the mutations in 21-Hydroxylase deficient patients.
• Bioinformatic analysis served as a pivotal tool in understanding functional consequences of the mutation's on the protein.

## 1 Data

**Fig. 1:**
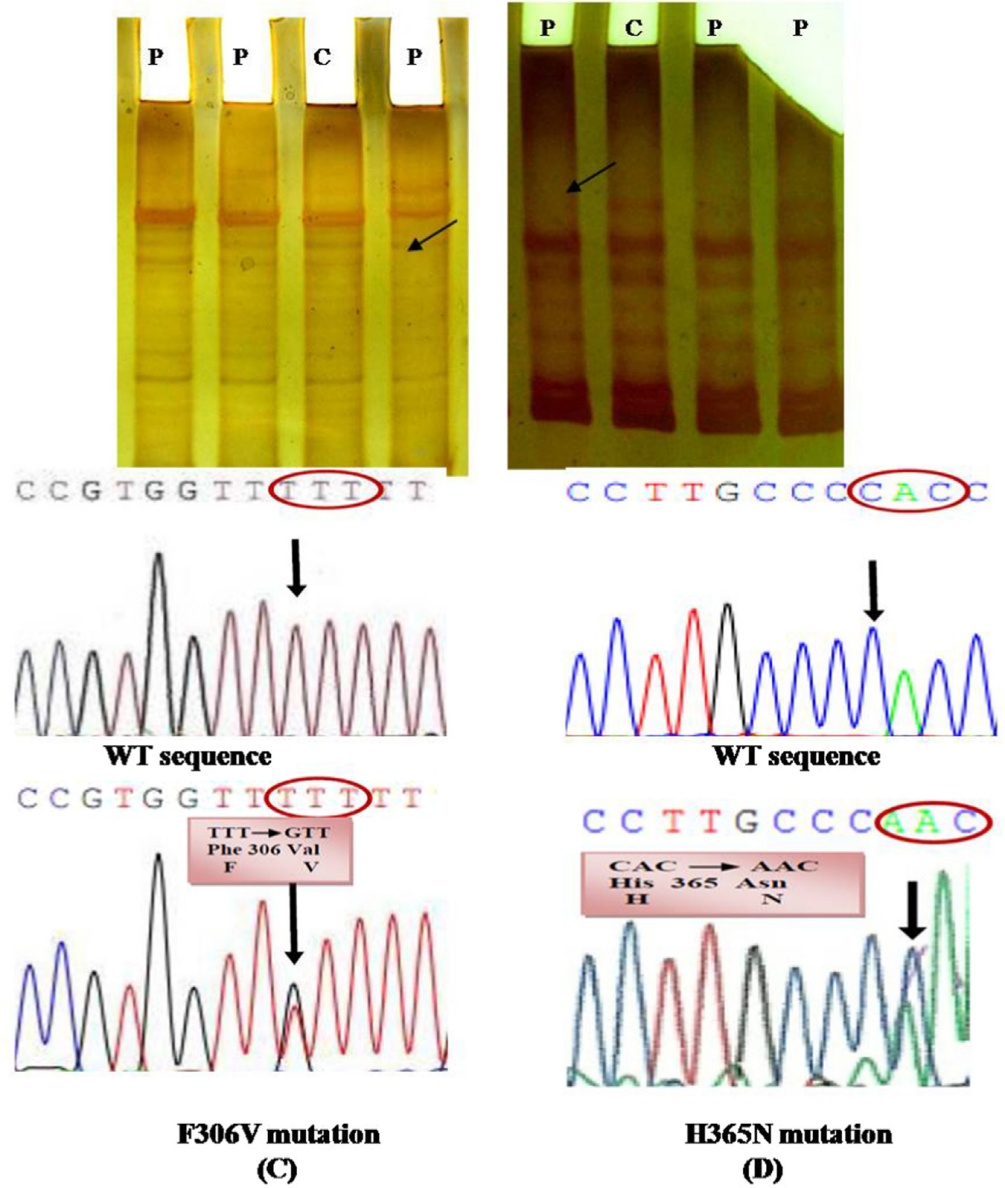
Detection of novel mutations by SSCP and DNA sequencing. (A) SSCP analysis of exon 7. N- Control, P -CAH patient. Arrow indicates the deletion in band. (B) SSCP analysis of exon 8. C- Control, P -CAH patient. Arrow indicates the deletion in band. (C) CYP21A2 gene sequence showing the Wild type sequence, CYP21A2 gene showing substitution of T to G in a heterozygous state at position 916 in cDNA resulting in a missense mutation at codon 306. (D) CYP21A2 gene sequence showing the Wild type sequence, CYP21A2 gene showing substitution of C to A in a heterozygous state at position 1095 in cDNA resulting in a missense mutation at codon 365.

**Fig. 2:**
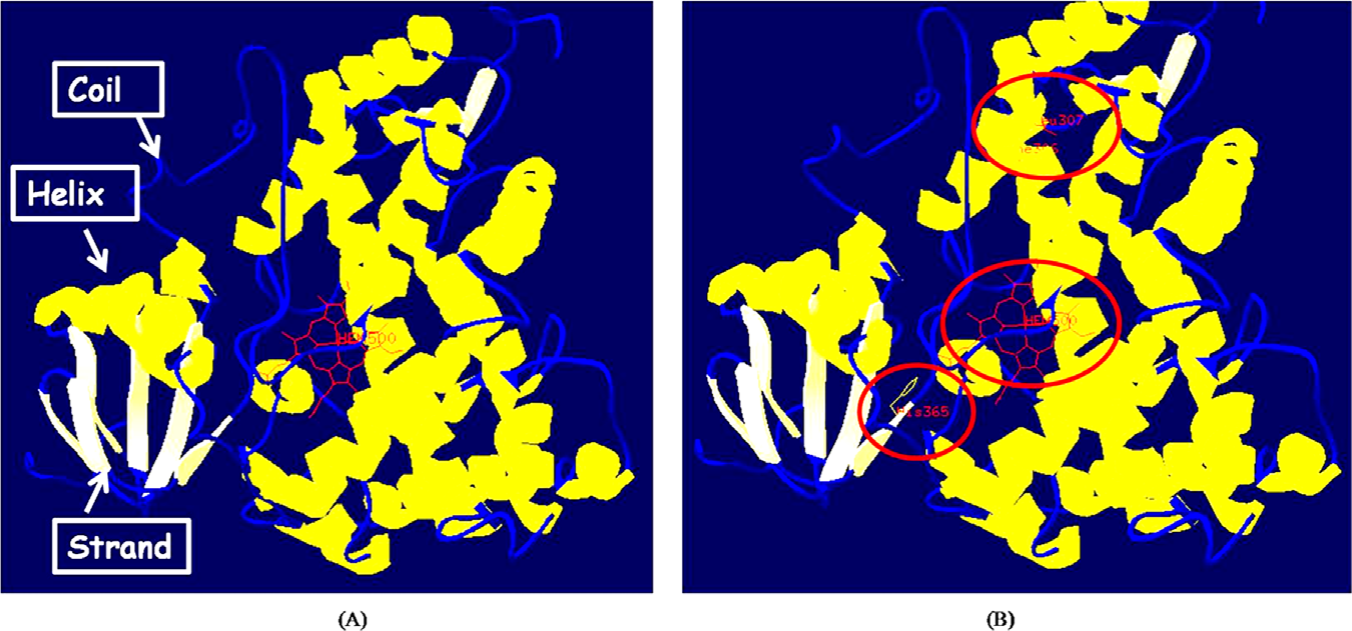
Ribbon diagram of human P450c21 as generated by Swiss PDB viewer. (A) Overall structure of the 3D model of CYP21A2 indicating helices, strands and coils in the protein structure. (B) 3D model structure of CYP21A2 indicating localization of heme, His365, Phe306 and Leu307 in the structure.

Congenital Adrenal Hyperplasia (CAH, OMIM #201910) is an inborn error of metabolism which describes a group of autosomal recessive disorder, characterized by the enzyme defects in the steroidogenic pathway [2]. Molecular analysis of CYP21A2 gene is of utmost importance to for the clinical diagnosis [3]. Mutations were identified in CYP21A2 gene by SSCP and subsequent DNA sequencing [1] (Fig. 1). Moreover, the functional characterization of the mutations is equally pivotal for diagnosis and subsequent genetic counseling. The sequence of the primers for the functional characterization of p.H365N, p.F306V and Double mutant (p.F306V, F306+T frameshift mutation) are listed in Table 1. Additionally, bioinformatic analysis acts as a complement to the *in vitro* characterization. SIFT, PROVEAN and PolyPhen were used to predict the effect of mutations on the 21-Hydroxylase protein (Table 2). The structure of the resultant mutant 21-Hydroxylase protein was analysed by HOPE (Have yOur Protein Explained) (Table 3). Overall structure of the 3D model of CYP21A2 was constructed using Swiss-PDB viewer indicating helices, strands and coils in the protein structure. Also, indicating localization of heme, His365, Phe306 and Leu307 in the. protein (Fig. 2).

**Table 1:** List of primers used for the site directed mutagenesis.

**p.F306V**	F – CCTCTCCTGGGCCGTGGTTgTTTTGCTTCACCACC
	R – GGTGGTGAAGCAAAAcAACCACGGCCCAGGAGAGG
**p.H365N**	F – CCTTAGCCTTGCCCaACCGCACCACACGGCC
	R – GGCCGTGTGGTGCGGTtGGGCAAGGCTAAGG
**Double mutant (p.F306V, F306+T frameshift mutation)**	F – CCTCTCCTGGGCCGTGGTTgTTTTtGCTTCACCACC
	R - GGTGGTGAAGCaAAAAcAACCACGGCCCAGGAGAGG

**Table 2:** List of the mutants and the in silico prediction of CYP21A2 mutations.

Mutation/polymorphism	Codon change	Prediction phenotype (Score)		
		SIFT	PROVEAN	PolyPhen
**F306V**	TTT → GTT	Affect protein function (0.01)	Deleterious (− 50420)	Probably damaging (0.996)
**H365N**	CAC → AAC	Affect protein function 0.01)	Deleterious (− 6.415)	Probably damaging (1.000)

**Table 3:** Severity prediction of the mutants by HOPE algorithm.

	Structure prediction by HOPE
**F306V**	Due to the smaller size of mutant amino acid in the protein as compared to wild-type, it caused an empty space in the core of the protein
**H365N**	Wild-type residue has interactions with ligand “heme”. The difference in properties of mutant and wild-type easily caused loss of interaction with the ligand heme. The new mutant residue was too small to make the multimer contacts in the protein structure.

## 2 Experimental design, materials and methods

### 2.1 SSCP: identification of mutations

All amplified exons of CYP21A2 gene were subjected to mutational analysis using SSCP as described by Sharma et al. [4].

### 2.2 Severity prediction

The output prediction score of the novel mutations was analyzed using software PolyPhen-2 (http://genetics.bwh.harvard.edu/pph). Pathological predictions were further confirmed by another computer algorithm PROVEAN (http://provean.jcvi.org/index.php) and SIFT (http://blocks.fhrc.org/sift/SIFT.html). In addition, two polymorphisms were also analysed for the severity prediction so as to check the efficacy of the computational programs. Effect of mutations over CYP21A2 protein structure was determined using software have your protein explained (HOPE) available at web server (https://www.cmbri.ru.nl/hope/) [5].

### 2.3 Homology modelling

Template model was human CYP21A2, previously generated as described by Robins et al. [6] and available at Protein data Bank (PDB) with ID 2GEG. It was based on the X-ray crystallographic cytochrome CYP2C5 (PDB code 1N6B), which shares 32% sequence identity and 50% sequence similarity. We determined the protein helices, sheets and turns along heme binding region in protein.
